# Alginate formulations with high loads of zebularine and retinoic acid promote tissue growth and innervation and induce extensive epigenetic repatterning

**DOI:** 10.1038/s41598-025-22528-8

**Published:** 2025-10-29

**Authors:** Paulina Słonimska, Jakub Baczyński-Keller, Rafał Płatek, Milena Deptuła, Maria Dzierżyńska, Justyna Sawicka, Oliwia Król, Paweł Sosnowski, Magdalena Koczkowska, Anna Kostecka, David K. Crossman, Michael R. Crowley, Piotr Sass, Ryszard Tomasz Smoleński, Piotr M. Skowron, Arkadiusz Piotrowski, Michał Pikuła, Sylwia Rodziewicz-Motowidło, Paweł Sachadyn

**Affiliations:** 1 Laboratory for Regenerative Biotechnology, Gdańsk University of Technology, Gdańsk, 80-233 Poland; 2 Laboratory of Tissue Engineering and Regenerative Medicine, Department of Embryology, Medical University of Gdańsk, Gdańsk, 80-211 Poland; 3 Division of Clinical Anatomy, Medical University of Gdańsk, Gdańsk, 80-211 Poland; 4 Department of Biomedicinal Chemistry, Faculty of Chemistry, University of Gdańsk, Gdańsk, 80-308 Poland; 5 Laboratory of Molecular and Cellular Nephrology, Mossakowski Medical Research Centre, Polish Academy of Sciences, Gdańsk, 80-308 Poland; 6 Department of Biochemistry, Medical University of Gdańsk, Gdańsk, 80-211 Poland; 7 3P-Medicine Laboratory, Medical University of Gdańsk, Gdańsk, 80-210 Poland; 8 Department of Biology and Pharmaceutical Botany, Medical University of Gdańsk, Gdańsk, 80-416 Poland; 9 Genomic Core Facility, University of Alabama at Birmingham, Birmingham, AL 35233 USA; 10 Division of Anatomy and Neurobiology, Department of Anatomy, Medical University of Gdańsk, Gdańsk, 80-211 Poland; 11 Department of Molecular Biotechnology, Faculty of Chemistry, University of Gdańsk, Gdańsk, 80- 308 Poland; 12 Department of Biochemistry, University of Physical Education and Sport, Gdańsk, 80-336 Poland

**Keywords:** Pharmacoregeneration, Epigenetic therapy, Tissue regeneration, Zebularine, Retinoic acid, Alginate carrier, Biological techniques, Biotechnology, Drug discovery

## Abstract

The study presents the development of a small-molecule epigenetic regenerative therapy that combines a demethylating agent, zebularine, with retinoic acid, acting as a transcriptional activator, and an alginate carrier. Subcutaneously injected formulations based on 2% sodium alginate containing high loads of zebularine (240 mg/ml) and retinoic acid (0.8 mg/ml) promoted regenerative responses in a mouse model of ear pinna punch wound involving the restoration of tissue architecture, the growth of nerve and vessel networks, and extensive alterations in gene methylation and expression profiles with no adverse effects in the animals. Among the remarkable changes in global gene methylation are those in neurodevelopmental genes. In vitro studies showed rapid discharge of zebularine but not retinoic acid from the alginate formulations. Live ultrasound imaging demonstrated gradual absorption of the subcutaneously injected alginate formulations, which may explain the in vivo activity of retinoic acid following subcutaneous administration. Cell culture tests exhibited no significant cytotoxicity of the alginate formulations. The simplicity of composition, preparation, and administration of alginate-based drug formulations is a distinctive advantage. The effective induction of regenerative response, together with a high safety profile of subcutaneously administered pro-regenerative alginate formulations, opens the way to testing further regenerative therapies for hard-to-reach lesions.

**Supplementary Information:**

The online version contains supplementary material available at 10.1038/s41598-025-22528-8.

## Introduction

 Small-molecule drugs, although effective in treating various conditions, remain uncommon in regenerative therapies. Nevertheless, pharmacological induction of tissue regeneration appears to be a promising direction. Epigenetic mechanisms control processes essential to the regenerative response, such as cell proliferation, differentiation, and pluripotency, which makes small-molecule epigenetic inhibitors potential tools that can be used to activate the regeneration process. Our previous research demonstrated that zebularine, a nucleoside inhibitor of DNA methyltransferases, induced ear pinna wound closure in mice, thereby showing that an epigenetic drug can enhance regenerative abilities^1^.

Tissue regeneration, understood as the restoration of lost tissue and its structure, may take several days or weeks, thus implying that effective regenerative therapies require extended treatment, which can be achieved with carriers that allow for gradual drug release. Hydrogel carriers exhibit properties that are functional for regenerative therapies. Hydrogels can be pre-loaded with small-molecule drugs or biologics, easily conform to the application site^2^, and gradually release the therapeutic load. Carriers that are desired for long-term contact with tissues should display excellent biocompatibility. Alginate meets these criteria, making it one of the most extensively tested biomaterials. Alginic acid is an anionic biopolymer of linear chains consisting of β-D-mannuronic acid and its C-5 epimer α-L-guluronic acid linked by 1–4-glycosidic bonds extracted from different brown algae species. It is soluble in water and forms stable hydrogels under mild conditions with high porosity in the presence of divalent cations, such as Ca^2+^, thanks to their interactions with the consecutive chains of α-L-guluronic acid (G-blocks)^3^. As supported by multiple experiments, alginate is biocompatible^4^. The Food and Drug Administration (FDA) has approved alginate for diverse medical applications, including dental impression materials, wound dressings, and Algisyl-LVR, for heart failure treatment, injected into the left ventricular wall, where it displays long-term stability^5^, as well as oral drugs to treat reflux^6^. Alginate is considered non-thrombogenic and non-immunogenic^5^, which may be beneficial for the immunoprotection of cell transplants. The absence of immunogenic responses to alginate can be attributed to the hydrophilic nature of the substance, which reduces its interactions with cell surface proteins^7^. In addition, foreign body reactions to alginate are associated primarily with impurities^8^. Alginate entered clinical trials involving implantations of alginate-encapsulated allogenic mesenchymal cells secreting glucagon-like peptide-1 into the brain for the treatment of intracerebral haemorrhage in stroke patients (NCT01298830), intraperitoneal transplantation of alginate-encapsulated porcine β-cell islets in patients with type 1 diabetes mellitus (NCT00940173, NCT01736228, NCT01739829), and intracranial implantation of alginate-encapsulated porcine choroid plexus in patients with Parkinson’s disease (NCT01734733). Interestingly, alginate alone may benefit the organism, as reported in the experiment where subcutaneously implanted Alzet pumps with sodium alginate prevented salt-induced hypertension in rats^9^.

As mentioned above, our previous work demonstrated that zebularine could activate tissue regeneration. Moreover, retinoic acid, a metabolite of vitamin A and a potent transcriptional regulator participating in regulating regeneration pathways^10^, synergistically enhanced the regenerative effect. The regenerative treatment involved the administration of 7 intraperitoneal injections of zebularine and 6 of retinoic acid^1^. Retinoic acid, a metabolite of vitamin A, is a transcriptional regulator that acts by binding its nuclear receptors, which target retinoic acid response elements in multiple genes^11,12^. Retinoic acid, known to be beneficial in various skin conditions, has also been approved for the treatment of acute promyelocytic leukaemia^13^. Retinoic acid signalling is known to induce regeneration in various tissues in amphibians and mammals^10^. Zebularine was tested for its anti-cancer activity as a DNA demethylating agent^14^, but it has never entered clinical trials. Later studies demonstrated that zebularine stimulated regeneration^1,15^ and is not harmful to animals even after long-term administration at high doses (400 mg/kg i.p. for 78 days)^16^. The concept of joint treatment with zebularine and retinoic acid assumes that zebularine-mediated demethylation releases epigenetic repression of genes essential for the regenerative response, including developmental genes silenced in adult organisms, while retinoic acid stimulates the activity of the derepressed genes. The synergistic effect of zebularine and retinoic acid on transcriptional activation was previously demonstrated experimentally in a cell culture model^1^.

In the present study, we combined the pro-regenerative activity of zebularine and retinoic acid with the potential of the alginate carrier to expand the possibilities of therapeutic applications. We developed a method to obtain alginate formulations with high loads of hydrophilic and hydrophobic drugs. In addition to the treatment’s safety and effectiveness, the simplicity of preparation and composition, demonstrated in our study, opens the way for further testing of alginate-based regenerative formulations with small-molecule drugs for hard-to-reach lesions.

## Results

### Preparation of alginate formulations with high loads of zebularine and retinoic acid

In our previous research, to induce the regenerative effects, we used zebularine dissolved in saline and retinoic acid in rape seed oil^1^. In the present study, we utilised the alginate carrier to prepare injectable formulations of both active substances, enabling the gradual release of the active compounds.

**Fig. 1:**
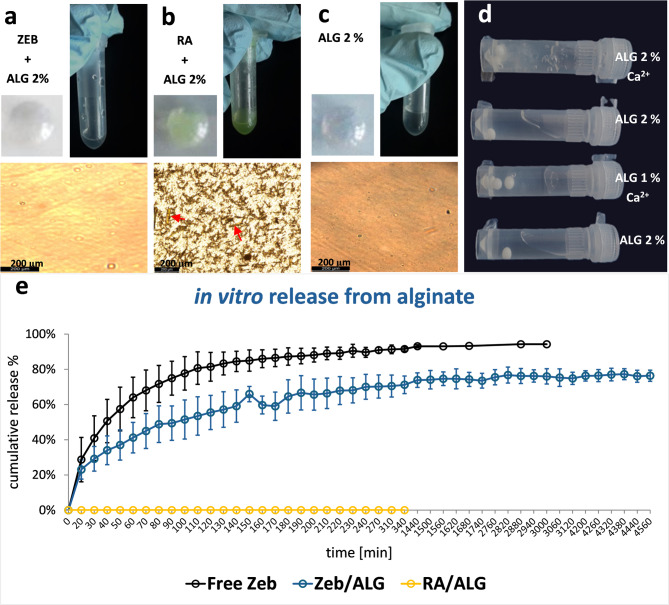
Alginate formulations of zebularine and retinoic acid. **a**) zebularine in 2% sodium alginate (240 mg per 1 ml), a 200 µl drop placed on a plastic dish (top left) in a tube (top right); and observed under a microscope (bottom); **b**) retinoic acid in 2% sodium alginate (4 mg per 1 ml), a 200 µl drop placed on a plastic dish (top left), in a tube (top right); and observed under a microscope (bottom, red arrows indicate tiny crystals of retinoic acid); **c**) for comparison, pure 2% sodium alginate, a 200 µl drop placed on a plastic dish (top left), in a tube (top right); and observed under a microscope (bottom); **d**) comparison of solidified Ca^2+^-crosslinked hydrogels with non-crosslinked injectable solutions of sodium alginate that were used to prepare the formulations with zebularine and retinoic acid (the solidified hydrogels do not flow down); e) In vitro release of zebularine and retinoic acid from non-alginate formulations; **e**) the percentage of cumulative release to PBS buffer from 2% sodium alginate formulation (non-crosslinked) containing 48 mg of zebularine (**ZEB**/**ALG**) or 0.8 mg of retinoic acid (**RA/ALG**) per 400 µl compared to free zebularine (10 mg/400 µl) (Free **ZEB**) measured in a dry heat diffusion cell. The data represent means +/- SD. The release experiments were performed in quadruplets, and those for free zebularine in duplicates.

Replacing saline with 2% sodium alginate was straightforward, given the excellent solubility of zebularine in aqueous solutions reported as 50 mg/ml ^1^. However, the treatment required seven intraperitoneal injections in saline at 500–1000 mg/kg body mass, corresponding to 70–140 mg of a collective zebularine dose per mouse weighing 20 g. Bead mill homogenisation facilitated the rapid preparation of injectable formulations containing high zebularine loads (Fig. 1a), precisely 48 mg per 200 µL, totalling 96 mg in two injections. For comparison, trials with Ca^2+^-crosslinked alginate hydrogels were unsuccessful, as such formulations rapidly solidified and were not injectable (Fig. 1d). The corresponding load of 240 mg of zebularine per 1 ml of 2% sodium alginate dramatically (almost five-fold) exceeded its solubility in aqueous solutions, allowing subcutaneous administration at a reasonable volume of approximately 200 µl per injection site in mice.

As previously reported, to enhance the regenerative effect of zebularine, retinoic acid was administered in rape seed oil in six doses at 8–16 mg/kg, corresponding to a collective dose of 0.96–1.92 mg per 20 g mouse^1^. Obtaining an injectable alginate formulation of retinoic acid, which is practically insoluble in water (0.21 µM − 63.1 ng/ml ^17^), seems more challenging than in the case of zebularine. A formulation containing 0.8 mg of retinoic acid in 200 µl of 2% sodium alginate (over 60,000 times the water solubility of retinoic acid^17^ was obtained using bead mill homogenisation, allowing a collective amount of 1.6 mg in two injections. The formulation was injectable and uniform under visual inspection (Fig. 1b).

### Microscopic examination of alginate formulations of zebularine and retinoic acid

Examination with a light microscope **(**Fig. 1a**)** did not identify any undissolved reagent residues (given that the image resembled that of the formulation of pure 2% sodium alginate (Fig. 1c), thus demonstrating the homogeneity of the alginate formulation with zebularine. In contrast, microscopic examination revealed fine, uniformly distributed crystals of retinoic acid **(**Fig. 1b**)**, invisible to the naked eye.

### In vitro release of zebularine and retinoic acid from alginate formulations

In vitro release was examined in a dry heat testing diffusion system in PBS buffer at pH 7.4 for alginate formulations of zebularine and retinoic acid. The formulation based on 2% sodium alginate gradually released zebularine **(**Fig. 1e**)**. The process commenced immediately after the formulation was placed in the donor chamber, reaching approximately 33% release within the first 40 min. Over the next 40 min, the cumulative release achieved approximately 50% and approached a plateau of around 70% by 4 h. Then, the process markedly slowed down, reaching a cumulative release of approximately 75% at 24 h and remaining at this level until the end of the experiment at 76 h. Considering the zebularine half-life of 508 h at 37 °C in PBS buffer at pH 7.0 ^1^, the analysis time was unlikely to impact the results. As expected, retinoic acid, practically insoluble in water^17^, showed no release from its alginate formulation within the experiment timeframe **(**Fig. 1e**)**.

### Effects of alginate formulations of zebularine and retinoic acid on cultured cells

Previous research in cell culture models demonstrated cytotoxic effects at elevated concentrations of zebularine^1,18^, and retinoic acid^19,20^ exerted on keratinocytes and fibroblasts. The present study tested the cytotoxic and cell viability effects of zebularine and retinoic acid alginate formulations. For the tests, the cultures of HaCaT keratinocytes and 46BR.1 N fibroblasts were treated with extracts obtained by incubating the cell culture medium for 24 h at 37 °C with an alginate formulation containing either zebularine (240 mg per 1 ml), retinoic acid (4.0 mg per 1 ml), or none. Treating cell cultures with extracts obtained by incubating the tested materials in a culture medium is used to model the interactions of materials with cells and tissues. ISO 10993-5:2009 guidelines recommend such an experimental setup.

**Fig. 2:**
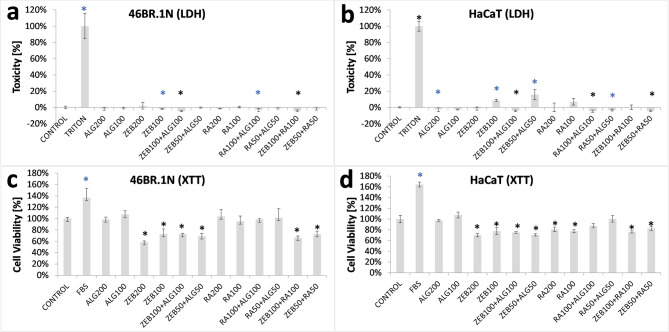
Effects of alginate formulations on cultured fibroblasts (46BR.1 N) and keratinocytes (HaCat) assessed with** (a**,** b) **cytotoxicity (LDH) and** (c**,** d) **cell viability tests (XTT). The cells were exposed to the extracts obtained by incubating alginate formulations in a DMEM HG medium. Each test was performed in 4 replicates; error bars represent the standard deviation (SD). Statistical analysis was carried out with the Kruskal-Wallis test followed by *post-hoc* analysis using the Conover-Iman procedure and the Bonferroni correction for multiple comparisons, assuming the corrected significance level of *p* < 0.0005; significant differences relative to the CONTROL were marked with blue and black asterisks corresponding to *p* < 0.05 and *p* < 0.0005, respectively. The treatments are denoted as follows: **CONTROL** - cells cultured in DMEM HG without serum; **FBS** - cells cultured in DMEM HG with 10% fetal bovine serum; **TRITON** - cells cultured in DMEM HG with 1% (v/v) Triton X100, a control corresponding to maximum releasable LDH activity; **ALG200** – 200 µl of extract from pure 2% sodium alginate formulation **ALG100** – 100 µl of extract from 2% sodium alginate + 100 µl of DMEM HG; **ZEB200** – 200 µl of extract from 2% sodium alginate containing zebularine (240 mg per 1 ml); **ZEB100** – 100 µl of extract from 2% sodium alginate containing zebularine (240 mg per 1 ml) + 100 µl of DMEM HG; **ZEB100**+**ALG100** – 100 µl of extract from 2% sodium alginate containing zebularine (240 mg per 1 ml) + 100 µl of extract from 2% sodium alginate; ZEB50**+ALG50** – 50 µl of extract from 2% sodium alginate containing zebularine (240 mg per 1 ml) + 50 µl of extract from 2% sodium alginate + 100 µl of DMEM HG; **RA200** – 200 µl of extract from 2% sodium alginate containing retinoic acid (4.0 mg per 1 ml); **RA100** – 100 µl of extract from 2% sodium alginate containing retinoic acid (4.0 mg per 1 ml) + 100 µl of DMEM HG; **RA100**+**ALG100 **– 100 µl of extract from 2% sodium alginate containing retinoic acid (4.0 mg per 1 ml) + 100 µl of extract from 2% sodium alginate; **RA50+ALG50** – 50 µl of extract from 2% sodium alginate containing retinoic acid (4.0 mg per 1 ml) + 50 µl of extract from 2% sodium alginate + 100 µl of DMEM HG; **ZEB100**+**RA100** – 100 µl of extract from 2% sodium alginate containing zebularine (240 mg per 1 ml) + 100 µl of extract from 2% sodium alginate containing retinoic acid (4.0 mg per 1 ml); **ZEB50**+**RA50** – 50 µl of extract from 2% sodium alginate containing zebularine (240 mg per 1 ml) + 50 µl of extract from 2% sodium alginate containing retinoic acid (4.0 mg per 1 ml) + 100 µl of DMEM HG.

No significant cytotoxic effects were determined in the cultured 46BR.1 N fibroblasts (Fig. 2a**)** and HaCaT keratinocytes (Fig. 2b**)**.

Reductions in cell viability by approximately 20–40% were observed in both 46BR.1 N fibroblasts **(**Fig. 2c**)** and HaCaT keratinocytes **(**Fig. 2d**)** after exposure to the extracts collected from the alginate formulations of zebularine and retinoic acid. These effects were significant in the keratinocyte cell cultures for all extracts containing zebularine, retinoic acid, or both, except for that with retinoic acid extract mixed with the extract collected from pure alginate formulations (Fig. 2d, RA50+ALG50). In the fibroblast cell cultures, the decreases were significant only for the extracts obtained from the formulations containing zebularine, either alone or with retinoic acid, but not those with retinoic acid alone (Fig. 2c, RA50+ALG50, RA100+ALG100, RA100, RA200). The effects of zebularine extracts on cultured cells correlate with the observations of zebularine release from alginate formulations in vitro **(**Fig. 1e**)**. While no retinoic acid release was detected in vitro **(**Fig. 1e**)**, the lowered keratinocyte viability **(**Fig. 2d**)** indicates that retinoic acid can be freed from its alginate formulations into the culture medium.

In conclusion, the reduced cell viability indicated that alginate formulations released zebularine and retinoic acid into the culture medium. The effect was moderate, considering the extreme zebularine concentration in the alginate formulations. The absence of cytotoxicity demonstrates a remarkable safety profile of the tested alginate formulations.

### Effects of alginate formulation of zebularine and retinoic acid on ear Pinna wound closure

**Fig. 3:**
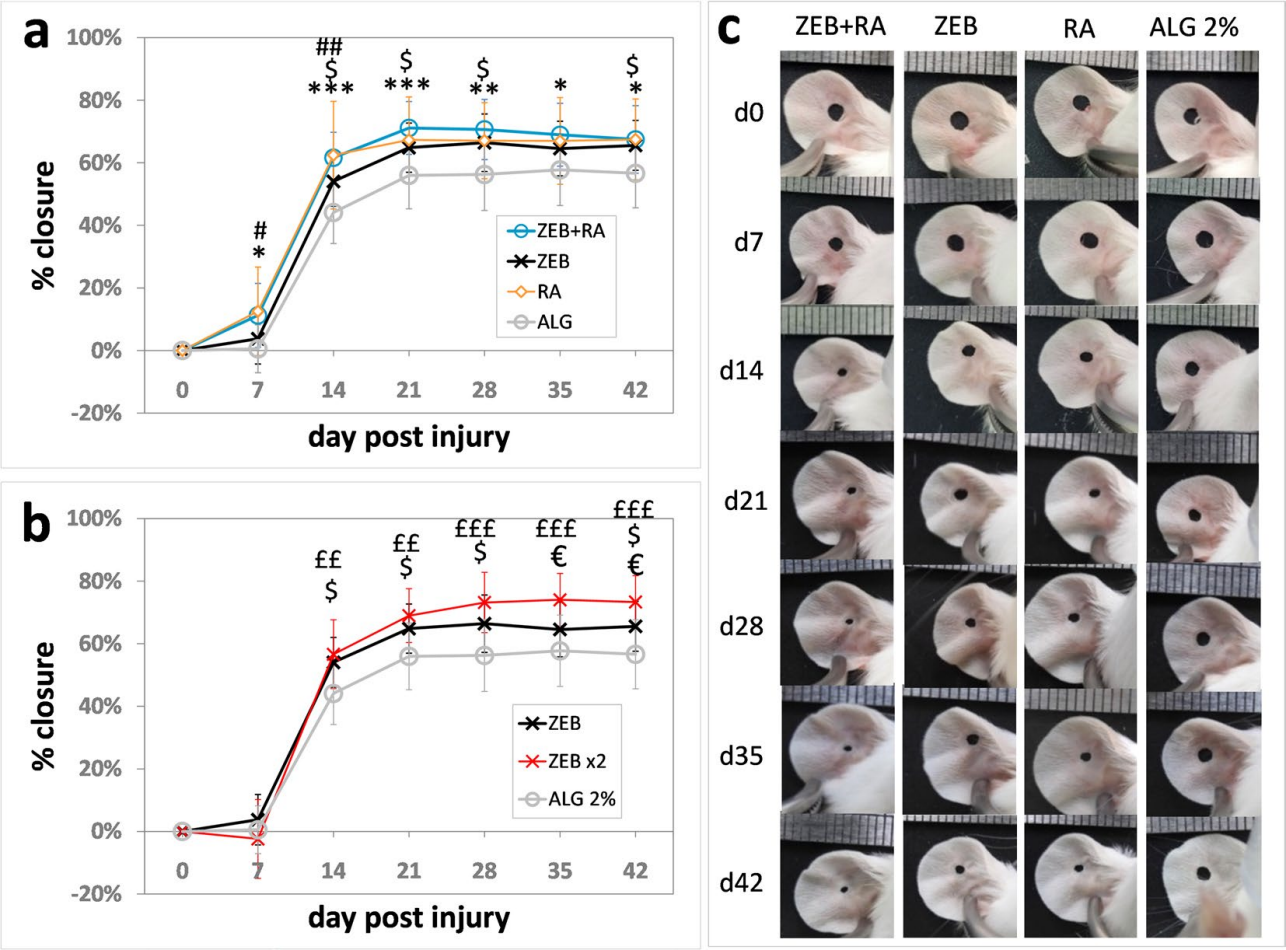
Progress of ear pinna hole closure in mice treated with the formulations of zebularine and retinoic acid in 2% sodium alginate.** a**,** b)** mean percentages of ear hole closure for six-mice experimental groups (*n* = 12 ears) receiving subcutaneous injections of alginate formulations on days 0 and 10 post-injury; error bars represent standard deviation. The data represent means of wound closure percentages calculated for each ear relative to the initial hole area according to the formula % closure = 1-a_dX_/a_d0_*100% where a_dX,_ and a_d0_ represent ear hole areas at a given time point and on day 0, respectively. **c)** representative photographs of ear pinnae. The treatments were designated as follows: **ZEB**+**RA** − 48 mg of zebularine in 200 µl of 2% sodium alginate + 0.8 mg of retinoic acid in 200 µl of 2% sodium alginate; **ZEB** − 48 mg of zebularine in 200 µl of 2% sodium alginate + 200 µl of 2% sodium alginate; **RA** − 0.8 mg of retinoic in 200 µl of 2% sodium alginate + 200 µl of 2% sodium alginate; **ALG** - two 200 µl portions of 2% sodium alginate; **ZEBx2** - doubled zebularine dose, two 200 µl portions of 2% sodium alginate each containing 48 mg of zebularine each. Statistically significant differences were determined with the two-tailed Mann-Whitney U test and indicated as follows: with asterisks * for **ZEB+RA** vs. **ALG**, dollar signs $ for **ZEB** vs. **ALG**, hashtags # for RA vs. ALG, euro signs € for **ZEBx2** vs. **ZEB** (doubled vs. single dose), and pound signs £ for **ZEBx2** vs.** ALG**. Single, double, and triple signs denote *p* < 0.05, *p* < 0.001, and *p* < 0.001, respectively.

Tests on rodents are widely used in preclinical research to assess the activity of drug candidates. Ear punch wound closure in mice goes beyond a model of cutaneous wound healing. It is an example of tissue regeneration involving not only skin but also cartilage, muscle, vessels, and nerve fibres. Tracking the regeneration progress in the ear pinna is straightforward. Unlike in vitro models, the regenerative process is not isolated from the circulatory, immune, and nervous systems. Determining the percentage of ear hole closure can be used to identify candidate drugs for promoting tissue regeneration^21^. In the present study, we applied the ear pinna model to test alginate as a carrier for regenerative drugs. The alginate formulations of zebularine and retinoic acid prepared as described above were subcutaneously administered to mice. For reference, analogous treatments were performed with sodium alginate alone, alginate with zebularine alone, and alginate with retinoic acid alone. The treatment was carried out immediately after the injury and repeated on day 10 post-injury. Each tested drug improved ear pinna hole closure compared to the pure sodium alginate control **(**Fig. 3a, c**)**. The combined administration of zebularine and retinoic acid had the more significant effects compared to the drugs used alone, and what is important, at all time points **(**Fig. 3a**)**. Doubling the dose augmented the regenerative response to zebularine **(**Fig. 3b**)**. These results were in agreement with those obtained for intraperitoneal administration, as reported previously^1^. Relatively high standard error values result from biological variation, typical in wound studies^22^.

The experiment showed that alginate was an effective carrier not only for hydrophilic zebularine, readily forming homogenous mixtures in 2% sodium alginate, but also for retinoic acid, almost insoluble in aqueous media, dispersed in the alginate formulation as fine crystals **(**Fig. 1b**)**. What is more, both zebularine and retinoic acid in alginate formulations induced pro-regenerative activity after subcutaneous administration. Although both drugs improved ear pinna hole closure compared to the carrier alone, the joint administration of zebularine and retinoic acid exerted significant effects at all time points (Fig. 1a). Therefore, further analyses focused on the combined treatment.

**Fig. 4:**
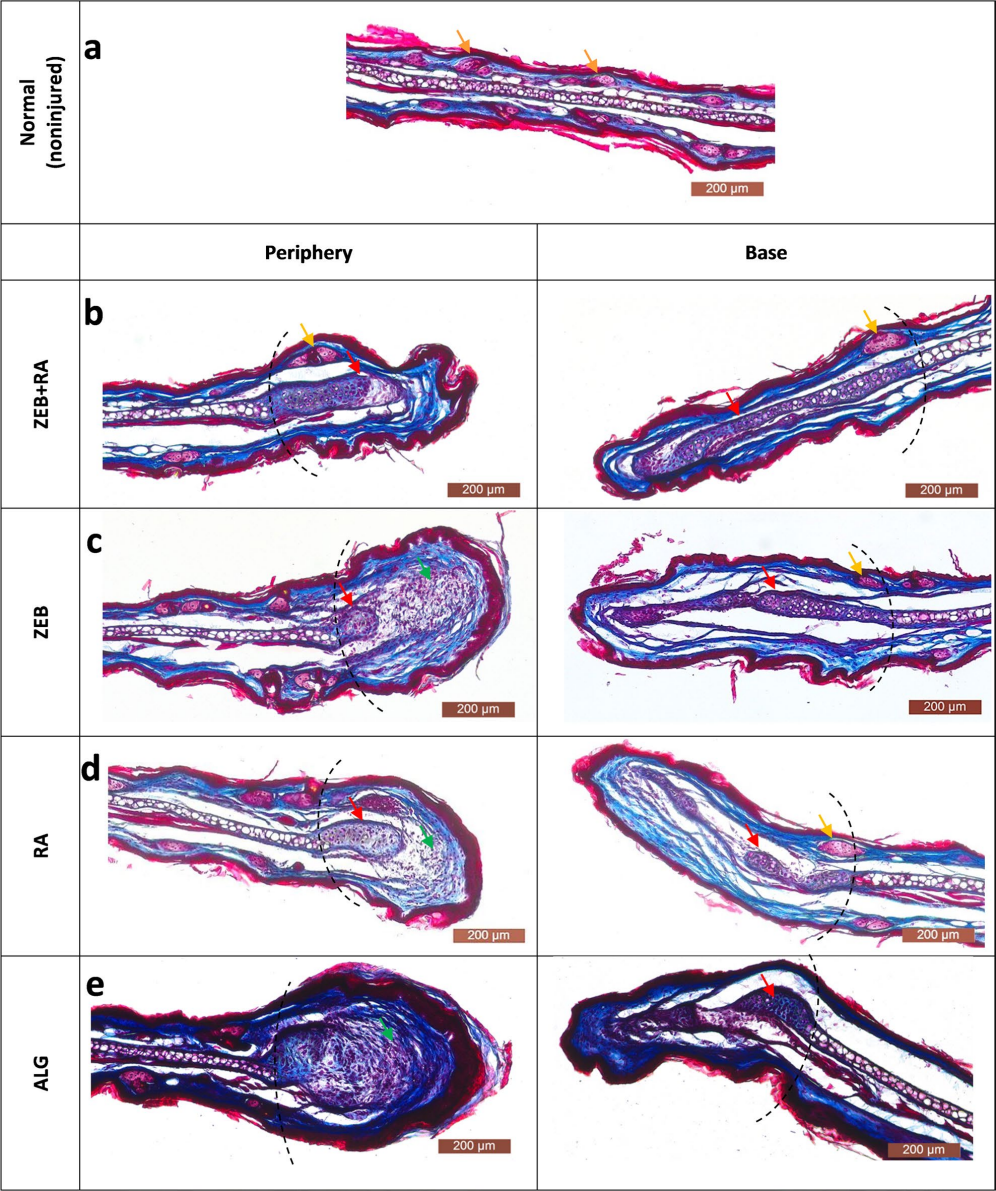
Histological examination of ear pinnae on day 42 post-injury stained with Masson trichrome.** a)** on the left: central part of noninjured ear pinna (normal tissue); on the right – diagram depicting horizontal projection (top) and coronal/sagittal plane of the sections (bottom); R – regrown/regenerated area; **b)** injured ear pinnae collected from the mice treated with alginate formulation with zebularine and retinoic acid - **ZEB+RA**; **c**) alginate formulation with zebularine alone - **ZEB**; **(d)** alginate formulation with retinoic acid alone - **RA**); and **(e)** 2% sodium alginate alone - **ALG**. The dotted lines mark the area of regrown tissue determined by comparing the ear hole diameters on days 0 and 42. The red and orange arrows mark the developing cartilage and adnexa, respectively. The green arrows indicate bulb-like structures with cells at the wound margin that were not found in **ZEB**+**RA**-treated mice. *Dermis* (collagen) stains blue; *epidermis* (keratin), muscles, cartilage, sebaceous glands, and nuclei stain purple. The “periphery” and “base” sections indicate the distal and proximal to the head, respectively.

Moreover, the post-mortem sections performed on day 42 post-injury disclosed no remains of the injected material in most (but not all) animals **(**Fig. 4c**)**. As demonstrated in *in vitro tests*, alginate formulation rapidly discharges zebularine (Fig. 1e). In the case of retinoic acid, subcutaneous absorption of the injected material may explain the release of the drug from the carrier and its activity in the animal’s body (this issue is expanded in the following section). Although administered in large quantities, the alginate carrier proved safe, as the animals betrayed no adverse effects. No signs of necrosis or irritation at the injection sites confirmed that the formulations were well tolerated under the skin, even though a zebularine concentration of 0.1 mg/ml, way lower than those achieved in the alginate formulations, was cytotoxic, as reported in cell models^1^.

### Histological examination of regenerated ear pinnae

**Fig. 5:**
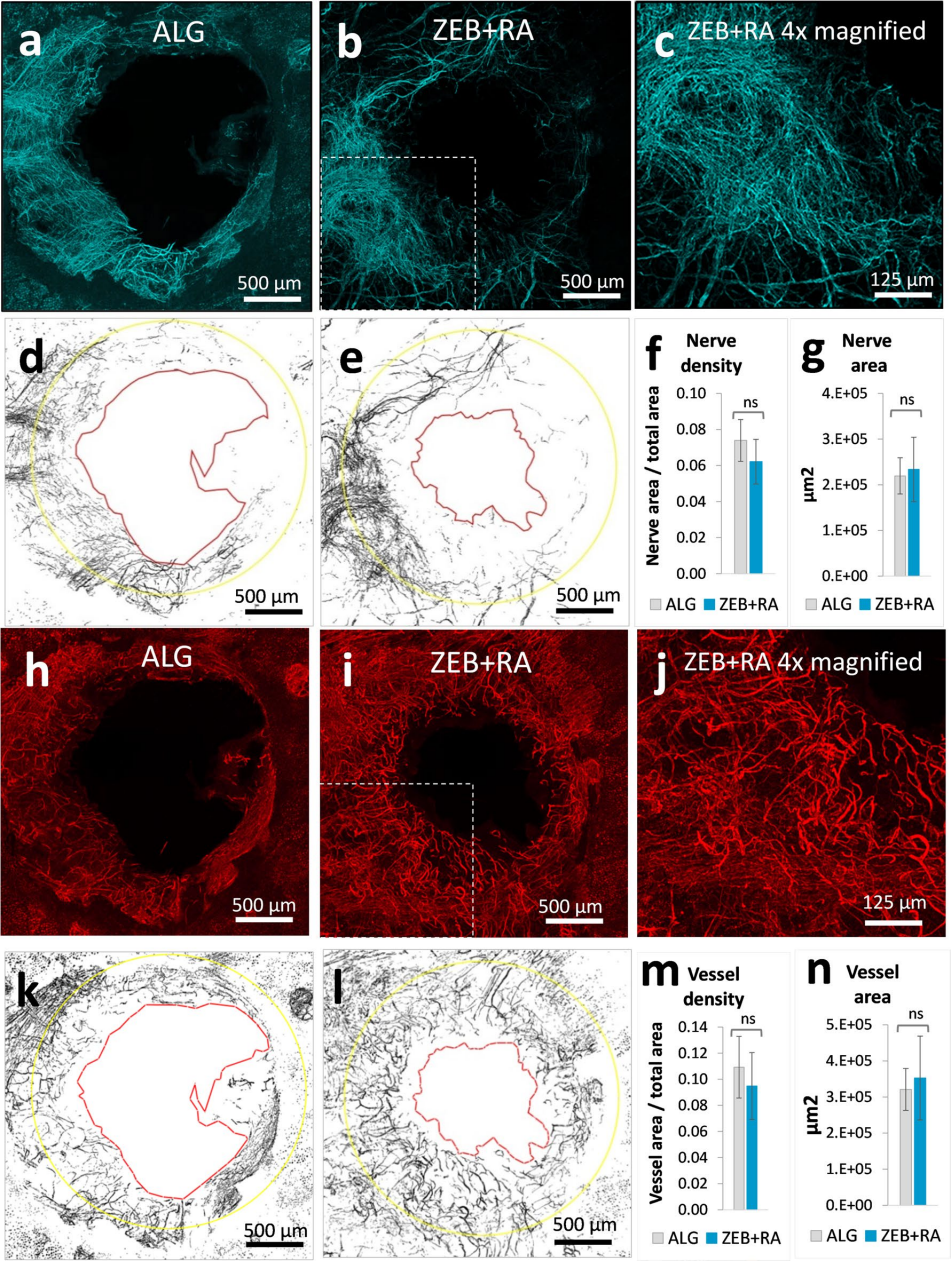
Peripheral nerves and blood vessels growing in regenerating mouse ear pinna on day 42 post-injury. Representative confocal images of the ear pinna outer aspect whole mount stained for neuron-specific anti-β-III-Tubulin (turquoise pseudocolour) and endothelium-specific anti-PECAM1 (red pseudocolour) antibodies collected from mice treated with **a**,** h)** pure 2% sodium alginate (**ALG**) and **b**,** c**,**i**,** j)** alginate formulations of zebularine (240 mg per 1 ml) and retinoic acid (0.8 mg per 1 ml) (**ZEB+RA**); binary images of **d**,** e**) nerve fibers and **k**,** l**) blood vessels with wound margins on day 42 post-injury marked with red lines and 2.4-mm of diameter yellow circles delineating the area of regrowth; densities of **f)** nerve fibers and **m)** blood vessels and total area of **g)** nerve fibers an **n)** blood vessels within the regenerating area. Means represent 5 ear pinnae from 3 mice treated with zebularine and retinoic acid (**ZEB+RA**, *n* = 5) and 6 ear pinnae from 3 mice treated with the carrier alone (**ALG**, *n* = 6). Error bars represent standard deviation. Statistical analysis was performed using the two-tailed heteroscedastic Student’s t-test.

Ear pinna hole closure allows for a preliminary assessment of the regeneration effect; however, it is vital to assess not only the extent but also the architecture of the restored tissues. A histological examination of the regenerating ear pinnae was performed on day 42 post-injury. The structure of the regrown tissue resembles that of a normal ear pinna, with the characteristic presence of the cartilage layer. At the wound edges, developing cartilage can be observed **(**Fig. 5b, c,d, e**)**. At the base side, the regrowing ear pinna has a thickness and structure similar to normal tissue, while it widens at the peripheral side. This widening of the peripheral wound edge is less pronounced in the mice treated with zebularine and retinoic acid than in the controls. The peripheral wound end takes a bulb-like structure **(**Fig. 5c, d,e**)**, except for the mice treated with the combination of zebularine and retinoic acid **(**Fig. 5b**)**. No cartilage can be distinguished in the bulb-shaped peripheral end in the controls instead, there is an intensive proliferation, and the wound edge is closed by a thick layer of connective tissue **(**Fig. 5e**)**. In contrast to the controls, skin appendages are found within the regenerating area in mice treated with zebularine and retinoic acid **(**Fig. 5b, c,d**)** similar to those in normal tissue **(**Fig. 5a**)**. Collagen density was similar in tissues treated with the combination of zebularine and retinoic acid and retinoic acid alone compared to the alginate control, while after treatment with zebularine were lower by 26–34% (Supplemental Fig. S1) The result suggests that retinoic acid may counteract zebularine-mediated decrease in collagen density. In principle, the characteristics of regenerated tissue architecture resemble those observed following zebularine administration in saline reported in the previous research^1^.

### Growth of nerves and vessels within the restored tissues

**Fig. 6:**
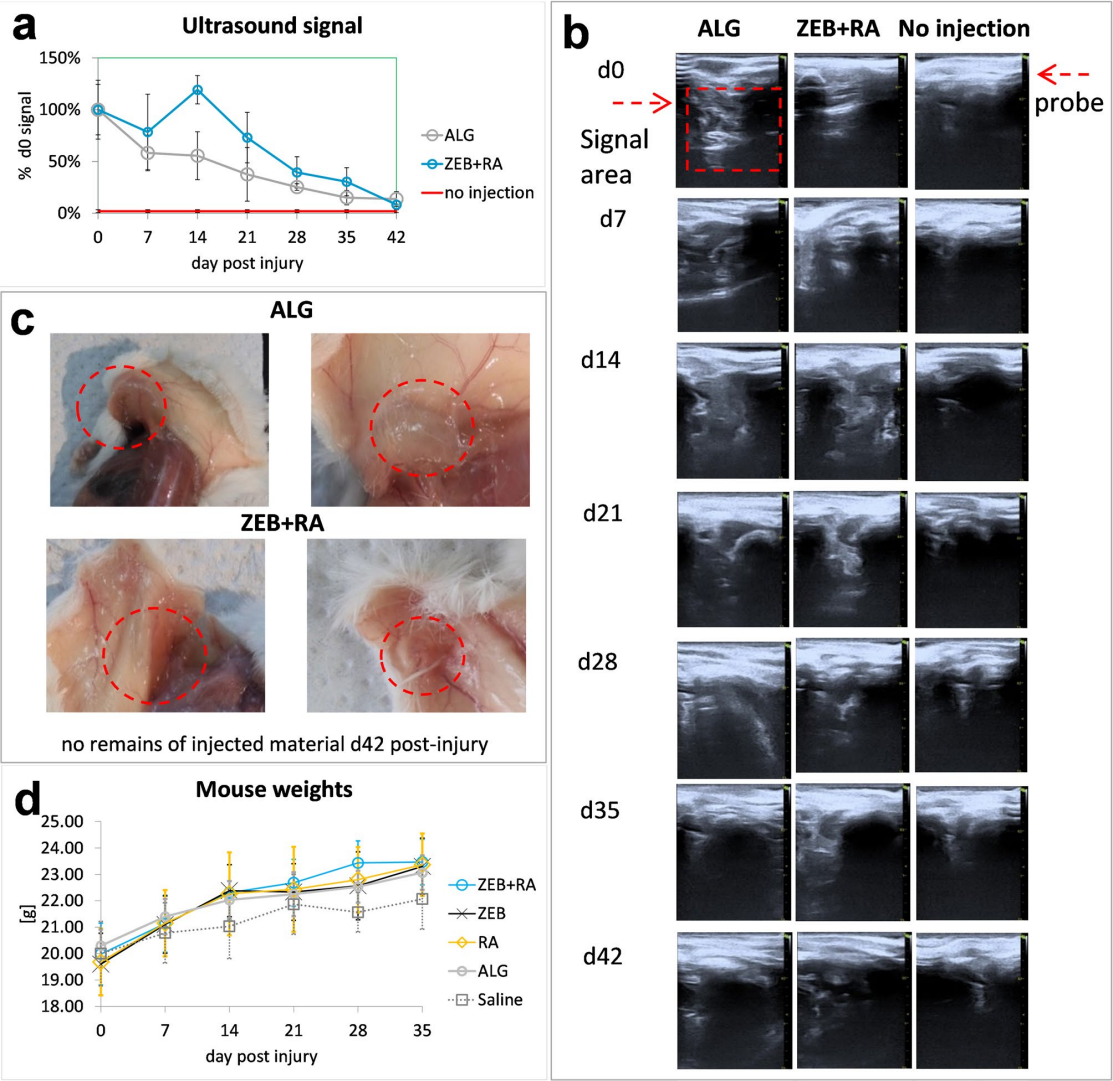
Live imaging of subcutaneously injected alginate-based formulations. **(a)** ultrasound signal from subcutaneous alginate formulations shown as a percentage of the signal measured on day 0. Ultrasound signals from alginate formulations were determined from 5–8 images. Each treatment was conducted on three mice (*n* = 3); error bars represent the standard error of the mean (SEM). No significant differences between the groups were found. **ALG** − 400 µl of 2% sodium alginate; **ZEB+RA** −48 mg of zebularine in 200 µl of 2% alginate sodium alginate and 0.8 mg in 200 µl of 2% sodium alginate; no injection - non-injected mice; **(b)** representative ultrasound images; **(c)** post-mortem sections on day 42 post-injection; **(d)** mouse weights in the course of the treatment with alginate formulations of zebularine and retinoic acid compared mice receiving saline injections; each mean represents 6 mice (*n* = 6); the Kruskal-Wallis test showed no significant differences between the tested groups.

Extensive networks of peripheral nerves and blood vessels supply the ear pinna^21,23^. The importance of blood supply in tissue regeneration is well understood; however, innervation is also critical, as observed in microsurgical denervation experiments on skin and ear pinna wounds^24–27^. In our experiment, nerve-specific immunostaining revealed that nerve fibres penetrated the regenerating ear pinna tissue in mice treated with zebularine and retinoic acid administered subcutaneously in alginate formulations **(**Fig. 6b, c,e**)** and in the controls receiving the carrier alone **(**Fig. 6a, d**)**. Our analysis identifed no significant difference in nerve fibre density in mice receiving the treatment compared to the controls in the circles of 2.4 mm in diameter surrounding the wounds, constituting the area covering the primary injury site (2 mm in diameter) and the peri-injury area affected by the wound healing and remodelling processes **(**Fig. 6f**)**. Although mice receiving the treatment showed an improved ear hole closure and therefore had more newly grown tissue, the total area of regrowing nerve fibres exhibited no difference compared to the controls. **(**Fig. 6g**)**. The outcomes indicate that the epigenetic treatment, while increasing the area of restored tissue, leads to re-innervation resembling that observed in the controls.

Staining with an endothelial cell-specific antibody showed dense networks of blood vessels supplying the regenerated tissues **(**Fig. 6h, i,j, k,l**)**. As in the case of nerve fibres, there were no significant differences in the densities and the total area of the regrowing vessels between the mice after the epigenetic treatment and the controls at day 42 (Fig. 6m, n). Interestingly, the fine nerves and vessels penetrating the regenerating area do not tend to align as the main ones do in the intact ear pinna^23^.

### Biocompatibility – the fate of subcutaneously injected alginate formulations

The post-mortem sections identified no presence of the injected material **(**Fig. 4c**)**, but whether its elimination was rapid or gradual remained unknown. Live-tracking of the alginate formulations injected under the skin of mice using ultrasonography was performed to address this issue. The presence of the injected material was determined by a comparative examination with mice that did not receive injections. The analysis revealed a gradual decrease in the ultrasound signals from the alginate formulations under the skin, indicating absorption **(**Fig. 4a, b**)**.

Post-mortem sections showed no necrosis in the contacting tissues despite huge concentrations of zebularine in the injected formulations **(**Fig. 4c**)**. In addition to no loss in body weight following the treatment **(**Fig. 4d**)**, the data confirm excellent biocompatibility of the tested alginate formulations.

### Gene methylation re-patterning and transcriptome changes in regenerating ear pinnae

**Fig. 7:**
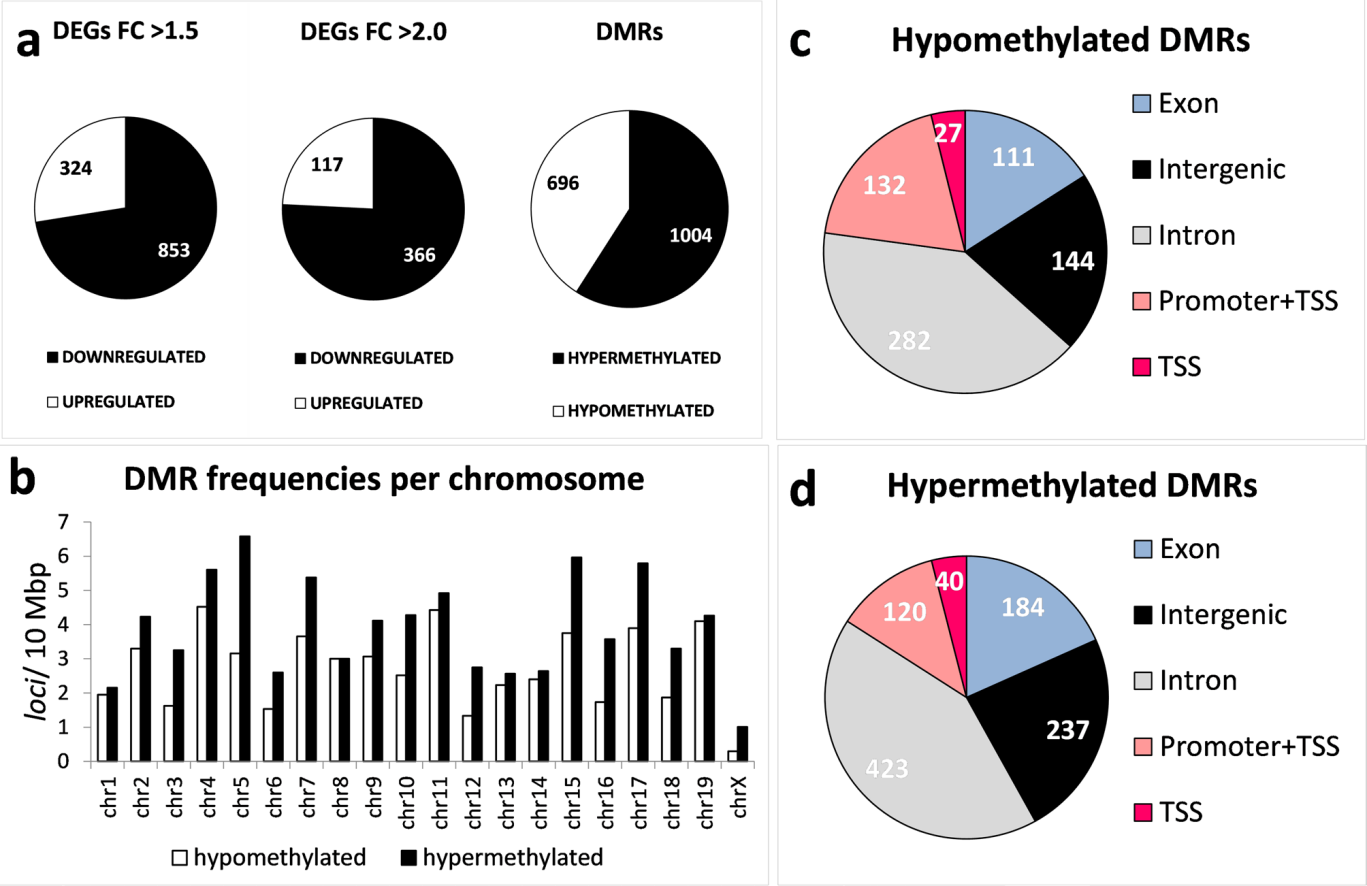
Genome-wide changes in gene expression and DNA methylation in regenerating ear pinnae in response to zebularine and retinoic acid administered in 2% sodium alginate, determined using RNAseq and BSseq. Gene expression and DNA methylation were examined in 3-mm tissue rings surrounding 2-mm wounds excised from ear pinnae 7 days after the injury and administration of zebularine and retinoic acid. The tissue samples for RNAseq and BSseq were obtained in independent experiments. Both the RNASeq and BSseq results represent data obtained from 6 mice (*n* = 6). **(a)** counts of differentially expressed genes (DEGs) displaying at least a 1.5-fold or a 2-fold change in expression and the numbers of differentially methylated regions (DMRs) in response to zebularine and retinoic acid treatment; **(b)** chromosomal distributions of DMRs; **c**,** d)** functional annotation of DMRs.

Genome-wide changes in transcription and DNA methylation following zebularine and retinoic acid treatment were determined using RNAseq and BSseq, respectively. The tissue surrounding wounds (3-mm rings) in the ear pinnae was collected 7 days after the injury, and subcutaneous injections of zebularine and retinoic acid in alginate formulations or of the carrier alone. This time point was selected to analyse the critical, early responses to the treatment, early but late enough to record DNA methylation re-patterning. Zebularine-mediated demethylation requires its incorporation into DNA, followed by cell proliferation during the growth phase of wound healing^1^, which typically begins on day 7 post-injury. The comparison of data from mice receiving treatment with those administered the carrier alone revealed substantial changes in gene expression and DNA methylation profiles. Hundreds of differentially expressed genes (DEGs) and differentially methylated regions (DMRs) were identified **(**Fig. 7a**)**. Supplemental File 2 presents the lists of the differentially methylated genes and the differentially expressed genes. The mean width of DMR and the mean distance of DMR to the nearest transcription start site were determined as 124 and 6124 bp, respectively. The counts of hypermethylated regions and downregulated genes decisively outnumbered those of hypomethylated and upregulated ones. No remarkable accumulation of DMRs on individual chromosomes was identified **(**Fig. 7b**)**. Most DMRs were located in the intronic and intergenic, while around 34–39% were in the regulatory and exonic regions **(**Fig. 7c, d**)**.

### Gene ontology analyses

As shown in Fig. 7, zebularine and retinoic acid target multiple genes. Therefore, to explain the mechanism of the regenerative response we observed, we focused on biological processes affected by the drugs. The gene ontology we performed revealed that both differentially expressed genes and those mapped to DMRs were significantly enriched in pathways crucial to regenerative processes **(**Table 1**)**. The pathways identified for the upregulated genes were related to keratinocyte differentiation and epidermis development, while those for the downregulated genes were associated with muscle cell differentiation and muscle tissue development **(**Table 1**)**. Development, differentiation, and morphogenesis are remarkable for the methylome re-patterning processes, as indicated by the enrichment of genes mapped to DMRs. In particular, “nervous system development” (GO:0007399) is the pathway identified for both the hypo and hypermethylated genes **(**Table 1**)**. The hypomethylated genes, i.e., those potentially activated in the wound margins, showed enrichment in those associated with the Wnt pathway and epithelium development. Notably, “epidermis development” was the signalling pathway also identified for the upregulated genes. The genes related to the remarkable pathways are listed in Supplemental File 3.

**Table 1:** Biological processes associated with the genes enriched in differentially expressed genes (DEGs) and genes mapped to differentially methylated regions (DMRs).

Upregulated in ZEB + RA-treated mice (3 mm, FC > 2, *p* < 0.05)	raw *p*-val	FDR
keratinocyte differentiation (GO:0030216)	6.02E-10	9.35E-06
epidermal cell differentiation (GO:0009913)	1.55E-09	1.20E-05
epidermis development (GO:0008544)	1.55E-07	8.02E-04
skin development (GO:0043588)	5.75E-07	2.23E-03
biological process involved in interspecies interaction between organisms (GO:0044419)	2.02E-05	4.49E-02

Downregulated in ZEB + RA-treated mice (3 mm, FC > 2, *p* < 0.05)	raw p -val	FDR
muscle structure development (GO:0061061)	5.08E-10	7.90E-06
muscle contraction (GO:0006936)	3.76E-07	8.35E-04
muscle cell differentiation (GO:0042692)	6.80E-07	1.32E-03
muscle tissue development (GO:0060537)	9.27E-07	1.44E-03
regulation of sequestering of calcium ion (GO:0051282)	7.58E-06	1.07E-02
muscle cell development (GO:0055001)	1.06E-05	1.26E-02
monoatomic ion transmembrane transport (GO:0034220)	3.89E-05	3.55E-02

Hypomethylated in ZEB + RA-treated mice	raw p -val	FDR
nervous system development (GO:0007399)	1.15E-07	2.55E-04
multicellular organism development (GO:0007275)	1.02E-07	2.65E-04
anatomical structure morphogenesis (GO:0009653)	8.02E-07	9.58E-04
neuron differentiation (GO:0030182)	1.47E-06	1.27E-03
neuron development (GO:0048666)	9.15E-06	4.74E-03
Wnt signalling pathway (GO:0016055)	1.25E-05	5.25E-03
embryonic morphogenesis (GO:0048598)	1.52E-05	6.07E-03
nitrogen compound metabolic process (GO:0006807)	2.49E-05	7.91E-03
regulation of cell differentiation (GO:0045595)	2.77E-05	8.26E-03
cell morphogenesis involved in neuron differentiation (GO:0048667)	2.72E-05	8.30E-03
positive regulation of developmental process (GO:0051094)	4.39E-05	1.18E-02
epithelium development (GO:0060429)	5.09E-05	1.30E-02
tissue development (GO:0009888)	1.07E-04	2.24E-02
neuron projection morphogenesis (GO:0048812)	1.08E-04	2.25E-02
animal organ development (GO:0048513)	1.33E-04	2.57E-02
programmed cell death (GO:0012501)	1.65E-04	3.05E-02
tissue morphogenesis (GO:0048729)	1.91E-04	3.40E-02

Hypermethylated in ZEB + RA-treated mice	raw p -val	FDR
nitrogen compound metabolic process (GO:0006807)	9.56E-06	8.74E-03
nervous system development (GO:0007399)	1.17E-05	1.01E-02
carbohydrate catabolic process (GO:0016052)	2.16E-05	1.68E-02

FDR correction (false discovery rate) was calculated using the Benjamini-Hochberg procedure.

FDR correction (false discovery rate) was calculated using the Benjamini-Hochberg procedure.

## Discussion

As reported in our previous research, multiple intraperitoneal injections of zebularine in saline and retinoic acid in oil induced tissue regeneration in the mouse ear pinna^1^. In the present study, we induced tissue regeneration with two subcutaneous injections of zebularine and retinoic acid in 2% sodium alginate. Subcutaneous administration is a safe and effective option, particularly when compared to the intraperitoneal route. In contrast, many drugs available on the market are administered subcutaneously^28^. The regenerative effect manifested in wound closure, tissue architecture restoration, and nerve fibres and blood vessel growth.

Significant alterations in methylation and expression status in several hundred genes in the regenerating tissues revealed the extensive scale of epigenetic repatterning underpinning the regenerative process. Nervous system development emerged as one of the remarkable pathways associated with the genes that were differentially methylated and differentially expressed in response to the regenerative treatment. This finding relates to the observed innervation of newly grown tissues. The repatterning of developmental genes, especially those involved in the nervous system, correlates with our previous findings on the induction of neurodevelopmental genes in ear pinnae regenerating in response to zebularine^1^. These results also correspond with the hypomethylation of developmental genes observed in models with enhanced regenerative potential^29–31^. Nerve dependence in regeneration has long been recognised in amphibians^32^, although it has been observed in mammals^24–26,33^, and still remains an understudied aspect. Our observations point to the role of neurodevelopmental genes in regenerative response. The genes hypomethylated in response to zebularine and retinoic acid treatment were also enriched in those involved in Wnt signalling, the activation of which is characteristic of regeneration^34^. Epithelial and muscle differentiation were identified as the significant biological processes connected with the genes differentially expressed in the regenerating ear pinna. The transcriptional induction of epidermal differentiation genes aligns with the occurrence of re-epithelialisation, an essential part of wound closure. The suppression of genes involved in muscle differentiation may indicate that this process is inhibited at this stage to orchestrate the overall regeneration process. Notably, a recent study by Shah et al. demonstrated that zebularine-mediated derepression improved epithelial wound healing in organ-cultured corneas from diabetic patients in a scratch assay^15^. Thus, an independent laboratory addressed our expectation that zebularine could promote regeneration in organs other than the ear pinna and organisms other than mice. What is more, Shah et al. identified the critical role of Wnt signalling in the mechanism of diabetic corneal healing, which corroborates the demethylation of Wnt pathway genes observed in our model. Our study highlights genes and pathways induced by zebularine, thus providing insight into the mechanism of zebularine-promoted regeneration. However, it should be stressed that epigenetic drugs by their nature target multiple genes, which makes elucidating their mechanism of action challenging.

Alginate proved to be an effective carrier for both hydrophilic zebularine and highly hydrophobic retinoic acid. Applying a bead mill enabled the rapid and convenient preparation of injectable formulations with extremely high loads of the active substance (240 mg of zebularine per 1 ml of 2% sodium alginate). The high doses of active compounds in the alginate formulations exerted no adverse effects on the contacting tissues; however, zebularine at elevated concentrations reduces cell viability and is cytotoxic, as previously reported^1,18^. In the cell model, the alginate formulations displayed no significant cytotoxicity but moderately decreased viability of keratinocytes and fibroblasts, thus indicating the release of zebularine and retinoic acid in cell culture conditions. The alginate carrier alone exhibited neither cytotoxicity nor an effect on cell viability.

As expected, the alginate formulation allowed for the rapid discharge of hydrophilic zebularine; nevertheless, the high amounts of the released substance deserve attention. In contrast, though it forms a homogeneous mixture with 2% sodium alginate solution, highly hydrophobic retinoic acid did not dissolve but formed fine crystals dispersed in the alginate formulation. However, both zebularine and retinoic acid activated regenerative responses in mice following subcutaneous injections in alginate formulations. Live ultrasound imaging in the animals showed gradual elimination of subcutaneously injected alginate formulations. The absorption of the carrier most likely explains the regenerative action of retinoic acid administered in the alginate formulation. This observation is important because, although alginate is known for its biodegradability in the body^35–37^, the fate of alginate in mammalian organisms has not been extensively studied.

The synergistic actions of zebularine and retinoic acid, as previously demonstrated^1^, underscore the benefits of combined treatment. Nevertheless, we demonstrated that drugs in the alginate carrier exhibited activity when applied combined or separately. This may be a helpful clue for researchers interested in testing alginate preparations with zebularine or retinoic acid alone, not exclusively in regenerative treatments.

### Final conclusions

Our study demonstrates the application of alginate in epigenetic regenerative therapy as a small-molecule drug carrier for subcutaneous administration, a route gaining increasing interest from researchers and the pharmaceutical industry^28^. Of note is that subcutaneous administration of alginate has rarely been tested, specifically not as a small-molecule drug delivery vehicle. Unlike methods using multi-component and covalently modified polymers, our approach offers simplicity of composition and preparation, translating into repeatability, versatility, and safety.

Our observations indicate that both hydrophilic and hydrophobic drugs can be safely administered in alginate formulations in subcutaneous injections following their release. The high load of active substance absorbed by the carrier, enormously exceeding its solubility in aqueous solutions (over sixty thousand-fold in the case of retinoic acid), deserves particular attention. Even if not released in vitro, hydrophobic compounds may penetrate tissues following the disintegration and absorption of the injected alginate carrier. This finding is of particular importance for testing compounds with limited solubility in water without using dimethylsulfoxide (DMSO), which is known to cause critical interference with essential life processes^38^.

Our experiments demonstrated that subcutaneously injected alginate formulations loaded with zebularine and retinoic acid promoted ear pinna regeneration in mice. Notably, the process involved the growth of nerves and vessels. Remarkable are the alterations in methylation and expression status of several hundred genes observed in the regenerating tissues, specifically the changes in methylation of neurodevelopmental genes. Due to the extensive gene expression remodelling involving the activation of developmentally silenced genes, epigenetic drugs can be powerful agents in triggering tissue regeneration.

## Methods

### Preparation of alginate formulations

Alginic acid sodium salt, manufactured from a species of brown algae, was purchased from Merck Cat. No. 71238, batch No. BCCD8789). The weight-average molar mass Mw and the number-average molar mass Mn, the authors reported, were 427 and 186 kDa, respectively, corresponding to a molecular weight distribution (MWD) of 2.3, thus classifying the material as a high-viscosity alginate. Mannuronic and guluronic acid contents were 39.6 and 60.4%, respectively, corresponding to a relatively low M/G ratio of 0.7, ranking the tested alginate among those with the highest guluronic content^39,40^.

The alginate carrier was prepared by suspending 20 mg of sodium alginate in 1 ml of water, followed by processing in a bead mill homogeniser (Bead Ruptor Elite, Omni International) using 2 mm porcelain beads at room temperature in three 30-s cycles at a speed of 4 m/s with 10-s breaks. The 2% sodium alginate solutions had a pH of 6.8. To prepare formulations with zebularine, 240 mg of zebularine (TCI, Cat. No. Z0022) was added to 1 ml of 2% sodium alginate and subjected to processing in a bead mill homogeniser by three 30-s cycles with 10-s breaks at 4 m/s using 2 mm porcelain beads. The alginate formulations with retinoic acid were obtained using the same protocol, except 4 mg of all-trans-retinoic acid (TCI, Cat. No. R0064) was added per 1 ml of 2% sodium alginate. For comparison with non-crosslinked solutions of sodium alginate, Ca^2+^-crosslinked alginate hydrogels were prepared by adding 10 µl of 7 M CaCl_2_ solution to 1 ml of 1 or 2% sodium alginate, followed by bead mill homogenising performed as detailed above.

### In vitro release

Release studies were performed using Phoenix DB-6 Dry Heat Diffusion System (Teledyne Hanson Research, the U.S.A.) equipped with 10 ml vertical diffusion cells with temperature maintained at 37 ± 0.1 °C. The system was stirred at a constant speed of 300 rpm. The Spectra/Por Dialysis membrane MWCO: 6–8,000 Da (Spectrum Labs, the U.S.A.) was used to separate the tested formulations from the receptor media. Zebularine in saline solution (10 mg per 400 µl), non-crosslinked alginate formulations of zebularine (48 mg per 400 µl), or retinoic acid (0.8 mg per 400 µl) were loaded on the membrane. The diffusion area was 107.5 mm 2. All release studies were performed in PBS buffer (pH 7.4). The experiments of release from alginate were performed in quadruplets, and the experiment for free zeularine in duplicates. A cumulative release profile of zebularine’s peak area vs. time was determined using the UHPLC system, using Phenomenex Kinetex 2.6 μm HILIC 100 Å, 100 × 2.1 mm column in an isocratic mode of 5 mM ammonium formate pH 3.2 in 90% (v/v) acetonitrile in water. The analysis of retinoic acid was performed on the UHPLC system, using Phenomenex Kinetex 2.6 μm C18 100 Å, 100 × 2.1 mm column in gradient mode of 5% to 100% B, where A is 0.1% of TFA in water and B is 0.1% of TFA in 80% (v/v) acetonitrile in water.

### Cell cultures

The cytotoxicity and the effect on cell viability of alginate formulations were tested on the cultured keratinocytes (HaCaT, DKFZ, Heidelberg) and fibroblasts (46BR.1 N, Sigma-Aldrich) treated with the extracts from the alginate formulations. The extracts were prepared by incubating the formulations (1 ml, prepared as detailed above) in 3.3 ml DMEM HG medium (Merck, Cat. No. D6429) for 24 h at 37 °C at a surface-to-volume ratio of 3 cm^2^/ml, followed by collection and centrifugation. The cells were seeded into 96-well plates (Corning, Cat. No. 353072), 5000 cells per well, in DMEM HG medium with 10% Fetal Bovine Serum (Merck, Cat. No. F9665), and allowed to attach for 24 h in standard conditions (5% CO_2_, 37 °C). After removing the medium, cells were treated for 24 h under standard conditions with extracts from alginate formulations, followed by LDH (Merck, Cat. No. 11644793001) and XTT (Merck, Cat. No. 11465015001) assays carried out in quadruplicate. Cells from the same passage were used. The analyses were conducted in accordance with ISO 10993-5:2009 guidelines.

### Animals

The study was conducted on female BALB/c mice aged 8–10 weeks at the beginning of the experiments. The animals were purchased from the Tri-City Academic Laboratory Animal Centre, where they were maintained. While our previous research proved that zebularine promoted ear hole closure in both females and males^1^, animals’ sex could be a confounder impacting wound closure. For this reason, to reduce data variation and, therefore, sample sizes, we decided to carry out the experiments on females in the present study. The animal experiment protocols were approved by the Local Ethics Committee for Animal Experimentation in Bydgoszcz, Poland (approval no. 51/2020). All experiments were performed in accordance with relevant guidelines and regulations. The study is reported in accordance with ARRIVE guidelines^41^. Mice were euthanised in a chamber with a gradually increasing carbon dioxide concentration up to 70%.

### Ear Pinna punch wound

After the mice were anaesthetised with isoflurane, through-and-through holes of 2 mm diameter were made in the centre of the ear pinna using a scissor-style ear punch (Hammacher Solingen; LOT FTC-15/8670/1). Prior to treatment, the animals were randomised into groups of six. Each group received subcutaneous injections of alginate formulations with zebularine, retinoic acid, their combination, or the carrier alone immediately after wounding (d0) and on day 10 post-injury (d10) in amounts indicated in the legend of Fig. 3. The subcutaneous injections were administered dorsally at the neck. The progress of wound closure was photographed weekly for 6 weeks, then followed by computer-assisted image analysis with ImageJ^42^. Prior to photographing, the mice were anaesthetised with isoflurane. The ear hole areas are listed in Supplemental File 1.

### Histology

On day 42 post-injury, after the mice were sacrificed, the ear pinnae were collected and fixed in 4% paraformaldehyde in 0.01 M phosphate-buffered saline (PBS), pH 7.4, at 4 °C. The tissues were then embedded in a tissue-freezing medium, cut into 10 μm sections using a Leica cryostat CM1520, and stained with Masson’s trichrome. Image acquisition was performed with a Leica DM IL LED DMC2900 microscope at 100× magnification.

### Collagen density

The density of collagen was calculated using the colour deconvolution method described by Ruifrok and Johnston from the images of tissue sections stained with Masson’s trichrome^43^. The procedure was carried out with the Colour Deconvolution 2 plug-in for Fiji software^44^. Images were loaded into Fiji. The stains were separated with the plug-in using Masson’s tri-chrome default settings. Then the colours were inverted. The densities of the blue dye representing collagen were quantified in randomly selected areas (9–12 per image) of *the dermis.*

### Immunofluorescence and confocal microscopy

On day 42 post-injury, the animals were sacrificed, and both ear pinnae per mouse were collected in 4% paraformaldehyde in 0.01 M phosphate-buffered saline (PBS), pH 7.4 and fixed at 4 °C overnight. Next, the ears with the higher degree of wound closure (left or right) from each mouse were selected, transferred to 0.01 M PBS, and dissected into outer and inner aspects (closer to the cheek). The outer aspects were used for staining and further analysis. Next, the visible remains of the cartilage layer were gently removed by scrubbing using a small spatula. Then, the ears were washed in 0.01 M PBS pH 7.4, 3 times for 5 min and then incubated for 1 h in a blocking buffer comprising 5% Normal Donkey Serum (NDS, Cat. No. 017-000-121, Lot 125.205) in 0.01 M PBS pH 7.4 with 0.5% Triton X-100 at room temperature. Next, the samples were incubated at 4 °C overnight with the primary antibodies diluted 1:300 in 1% NDS in 0.01 M PBS pH 7.4 with 0.1% Triton X-100: monoclonal mouse anti-Tuj1 conjugated with Alexa Fluor-647 (Biolegend, the U.S.A., Cat. No. 801210, Lot 367545) to detect neuron-specific class III ß-tubulin in nerve and rat anti-CD31/PECAM-1 to detect platelet endothelial cell adhesion molecule-1 as a pan-endothelial marker of vessels (BD Biosciences, U.S.A., Cat. No. 553369, Lot 015641). The next day, the samples were washed in 0.01 M PBS pH 7.4, 3 times for 5 min. Next, ears were incubated in the secondary antibody to visualise vessels - F(ab’)2 fragment of donkey anti-rat IgG conjugated with Alexa-Fluor-488 (Jackson Immunoresearch Europe Ltd., Ely, U.K., Cat. No. 712-546-150, Lot 157799) diluted 1:500 in 0.01 M PBS pH 7.4 with 0.1% Triton X-100, with gentle agitation, for 1 h at RT, followed by a washing step in 0.01 M PBS pH 7.4, 3 times for 5 min. Then, the ears were placed on a microscopic glass slide, covered with a VectaShield Vibrance mounting medium (Vector Laboratories, the U.S.A., Cat. No. H-1700-10), and a glass coverslip. Microphotographs of the ear pinnae were captured with the confocal microscope Zeiss LSM800 using ZEN 2.6 Software. Pictures of the wound area were taken using a 10× objective lens. Confocal scanning covered the middle 84 μm of the ear’s depth (8 optical slices) to capture all detectable nerve fibres. Photomicrographs were exported as TIFF files, and a maximum intensity projection of all z-stacks functions was used in ImageJ software to obtain one-plane images. The pictures were calibrated, subjected to uniform background subtraction to minimise the autofluorescence input, and changed to 8-bit images. Next, the wound edges were manually drawn to calculate the residual wound area. Then, the regenerated area was determined by subtracting the wound area from the original injury area (3 140 000 µm2). The nerve area was measured by autothresholding the 8-bit images. Finally, the density was calculated by dividing the nerve area by the regenerated area.

### Ultrasound examinations

Ultrasound imaging was used to investigate the fate of the alginate formulations injected subcutaneously in mice. The experiments used Vinno6 VET (VINNO Technology, Suzhou, China) and a 21 MHz linear probe. During the measurements, the mice were anaesthetised with isoflurane. Measurements were performed immediately after injection of the tested formulations and on days 7, 14, 21, 28, 35, and 42. After the final measurement on day 42, the mice were sacrificed, followed by post-mortem sections to confirm the absorption of subcutaneously injected alginate formulations. The experiment involved 3 mice that received zebularine (48 mg) in 200 µl of 2% sodium alginate and retinoic acid (0.8 mg) in 200 µl of 2% sodium alginate each. Three mice received only 400 µl of alginate alone, and three non-injected mice served as naïve controls. Photographs of the ultrasound signal from the area covering the nape and scapulae were exported as TIFF files, then scaled and transformed to 8-bit images using ImageJ. Next, the intensity auto threshold was applied to convert the images to binary masks, and the region of interest, the same for all the analysed images, was set based on the images from the non-injected naïve mice to cut off the non-specific signal from the ultrasound probe. The signals from the injected alginate formulations were computed from the corresponding areas on ultrasound images using 5 to 8 photographs per mouse per time point.

#### RNAseq

After euthanasia, ear pinnae for RNAseq were collected on day 7 post-injury, from 6 mice treated with zebularine and retinoic acid administered in alginate formulations and from 6 control mice receiving the carrier alone, as detailed above. After collection, the samples were immediately placed in liquid nitrogen and stored at −80°C. Total RNA was extracted from 3 mm rings surrounding the initial punch wound using RNeasy Mini kit (Qiagen). The tissues from a pair of ear pinnae from each animal were pooled prior to RNA extraction so that each RNA sample represented one mouse. The quantity of the extracted RNA was determined using the Qubit RNA assay (ThermoFisher Scientific), while the RIN (RNA integrity number) was determined using RNA Screen Tape Analysis on 4150 TapeStation System (Agilent). TruSeq Stranded Total RNA with Ribo-Zero Human/Mouse/Rat kit (Illumina) was used for library construction, followed by 5’ and 3’ adapter ligation. The cDNA libraries were sequenced in paired-end mode on the Illumina platform as a service provided by Macrogen. Sequencing data was converted into FASTQ format with Illumina’s bcl2fastq converter. All samples were trimmed using Trim Galore (version 0.6.7), and these trimmed reads were successfully aligned to Gencode’s GRCm39 Release M28 mouse reference genome using STAR (version 2.7.10a)^45^. Transcript assembly and estimation of the relative abundances were performed with HTSeq-count (version 2.0.2). Raw counts were then normalised using DESeq2 (version 1.36.0). The RNAseq data have been deposited in the ArrayExpress database under the accession number E-MTAB-13,957.

#### BSseq

The tissue samples were collected as detailed in the preceding section. Genomic DNA was extracted from 3 mm rings surrounding the initial punch wound using DNeasy Blood and Tissue kit (Qiagen). The tissues from a pair of ear pinnae from each animal were pooled prior to DNA extraction so that each DNA sample represented one mouse. The extracted DNA was quantified spectrophotometrically using the Varioskan Lux (ThermoFisher Scientific), and the DIN (DNA integrity number) was determined using Genomic DNA Screen Tape Analysis on the 4510 TapeStation System (Agilent). Reduced-representation bisulfite sequencing (RRBS) was performed by the Genomic Core Facility at the University of Alabama at Birmingham using the Ovation RRBS Methyl-Seq kit (Tecan Genomics), followed by sequencing DNA libraries on the NextSeq 500 platform (Illumina). Following Tecan’s Analysis Guide (https://github.com/nugentechnologies/NuMetRRBS), Trim Galore (version 0.6.7) was used to remove residual adapter sequences from the reads. Diversity trimming and filtering with their trimming script were then applied to the trimmed FASTQ files. These clean RRBS sequencing reads were aligned to the UCSC mm39 mouse reference genome using Bismark (version 0.22.1_dev)^46^. The aligned reads were then sorted using Samtools sort (version 1.15.1), following Bismark alignment, mapping, and sorting, methylKit (version 1.22.0) was used to identify CpG sites, normalise, and perform differential methylation analysis. The DMRs were identified with the BIOCONDUCTOR package bsseq using default parameters^47^. The BSseq data have been deposited in the ArrayExpress database under the accession number E-MTAB-13,955.

#### Ontological analyses

Genes displaying significant differences in expression (DEGs) with a mean fold change over 2.0 and genes mapped to the DNA regions showing significant differences in methylation (DMRs) between the treatment and control mice were included in ontological analyses (GO Enrichment Analysis) with PANTHER^48^ (GO Ontology database DOI: 10.5281/zenodo.10536401 Released 2024-01-17). Raw p-values were determined by Fisher’s exact test, following FDR (false discovery rate) correction calculated by the Benjamini-Hochberg procedure to filter out the results with FDR > 0.05.

### Statistical analysis

The statistical tests used are indicated in the figure legends. The statistical significance threshold was set at *p* < 0.05. The statistical computations were performed using XLSTAT (Addinsoft).

## Supplementary Information

Below is the link to the electronic supplementary material.

Supplementary Material 1

Supplementary Material 2

Supplementary Material 3

Supplementary Material 4

## Data Availability

The RNAseq and BSseq data generated during and analysed in the current study are available in the ArrayExpress repository under the accession numbers E-MTAB-13957 (https://www.ebi.ac.uk/biostudies/arrayexpress/studies/E-MTAB-13957?key=c4ff2289-bfa9-483c-a852-b6354e75ca99) and E-MTAB-13955 (https://www.ebi.ac.uk/biostudies/arrayexpress/studies/E-MTAB-13955?key=7349fc0e-fd4b-438e-a281-828e87391996).Wound closure data are listed in Supplemental File (1) Lists of differentially methylated and differentially expressed genes are available in Supplemental File (2) Genes related to remarkable pathways identified by gene ontology analyses are listed in Supplemental File 3.
