# Supplementary material for: Alginate formulations with high loads of zebularine and retinoic acid promote tissue growth and innervation and induce extensive epigenetic repatterning
Source: Sci Rep. 2025 Oct 29;15:37923. doi: 10.1038/s41598-025-22528-8 (PMC12572142; doi:10.1038/s41598-025-22528-8)
Supplement: Supplementary file 4 — Supplementary Material 4 [file 41598_2025_22528_MOESM4_ESM.docx]

**Supplemental Figure S1. Collagen densities in the ear pinna sections stained with Masson trichrome on day 42 post-injury (presented in Fig. 4).**

Statistical analysis was carried out with the Kruskal-Wallis test followed by *post-hoc* analysis using the Conover-Iman procedure and the Bonferroni correction for multiple comparisons, assuming the corrected significance level of *p* < 0.0083; the significantly different result was distinguished with an asterisk; error bars represent SD.
